# Supplementary material for: Fitness effects of mutations to SARS-CoV-2 proteins
Source: bioRxiv. 2023 Jun 6:2023.01.30.526314. Originally published 2023 Jan 31. Preprint. [Version 2] doi: 10.1101/2023.01.30.526314 (PMC9915511; doi:10.1101/2023.01.30.526314)
Supplement: Supplement 1 [file NIHPP2023.01.30.526314v2-supplement-1.pdf]

bioRxiv preprint doi: https://doi.org/10.1101/2023.01.30.526314; this version posted June 6, 2023. The copyright holder for this preprint (which was not certified by peer review) is the author/funder, who has granted bioRxiv a license to display the preprint in perpetuity. It is made available under aCC-BY 4.0 International license.

Supplementary appendix deriving relationship between fitness cost and ratio of expected to actual counts

With millions of SARS-CoV-2 sequences shared publicly, almost all mutations that are tolerated by the virus are observed dozens to hundreds of times. Where on the tree and how often on the tree we observe specific mutations has information about the effects of these mutations on viral spread. The mutation rate depends on the nucleotides involved and possibly on the sequence context and other viral determinants, but for the purpose of this derivation, we will assume the neutral rate μ is known. If the mutation is neutral, the total number of times the mutation is observed on the tree is μT, where T is the total length of the tree (assuming that the mutation never reached high frequency which is true for almost all mutations, particularly when mutations are counted on a per-clade basis relative too the clade founder as done above).

If a mutation reduces fitness, the lineages descending from branches on which this mutation happened will spread more slowly than those without this mutation. As a result, the down-stream subclades are smaller and more short lived, which in turn means that they will be less likely to be sampled and represented in the tree. To infer a mutation's effect on fitness, we need to calculate how the probability of observation depends on this fitness effect.

For a mutation to be represented in the tree, one of its descendants has to be sampled and sequenced. If the total number of descendants is w and the sampling fraction is ϵ, the probability that the mutation is present in the tree is

$$P = 1 - e^{-w\epsilon} \tag{1}$$

W is a random number that depends on the realization of the transmission process, which is commonly modeled by a branching process with birth rate b and death rate d. The death rate here corresponds to clearing an infection, the birth rate to onward transmission. The latter is affected by the fitness cost of the mutation.

To obtain insight how the probability of observing a lineage depends on parameters, we calculate the probability $p(w, T|t)$ that a lineage had an integrated size $w = \int_t^T k(t')\, dt'$, where t is the birth time of the lineage, T is the current time, and $k(t')$ is the size of the lineage at time t'. To calculate $p(w, T|k)$, we generalize it slightly to $p(W, T|k, t)$, where k is the number of individuals at the start time t. This quantity obeys the following "first-step" equation:

$$-(\partial_t - k\partial_w)p(w, T|k, t) = -k(b+d)p(w, T|k, t) + kbp(w, T|k+1, t) + kdp(w, T|k-1, t) \tag{2}$$

We will solve for the Laplace transform $\hat{p}(z, T|k, t) = \int_0^\infty dw\, e^{-wz} p(w, T|k, t) = \hat{p}^k(z, T|1, t)$. Using the following identity for the derivative of the Laplace transform

$$\int_0^\infty e^{-wz}\partial_w p\, dw = [e^{-wz}p]_0^\infty - \int_0^\infty p\partial_w e^{-wz}\, dw = 0 + z\int_0^\infty pe^{-wz} = z\hat{p} \tag{3}$$

and setting $k = 1$, we have

$$-\partial_t \hat{p}(z, T|t) = -(b+d+z)\hat{p}(z, T|t) + b\hat{p}^2(z, T|t) + d \tag{4}$$

This simplifies further to if we substitute $\phi(z, T|t) = 1 - \hat{p}(z, T|t)$.

$$\begin{aligned} \partial_t\phi(z, T|t) &= -(b+d+z)(1-\phi(z, T|t)) + b(1-\phi(z, T|t))^2 + d \\ &= -z - (b-d-z)\phi(z, T|t) + b\phi(z, T|t)^2 \end{aligned} \tag{5}$$

where it is important to note that the derivative is with respect to the first time point and the interval $T - t$ is shrinking with increasing t.

Constant birth and death rate

If the fitness effect of the mutation in question is detrimental and the overall population is constant (background $b_0 = d_0$), all mutant lineages will eventually die out and we can consider large $T - t$ and the long time asymptotic $\partial_t\phi(z, T|t) = 0$. Further define $b = b_0 - s$ and $d = d_0$ where s is the fitness cost of the mutation (so larger values indicate a greater fitness cost). The steady state generating function is then

$$0 = -z - (b-d-z)\phi(z) + b\phi(z)^2 \tag{6}$$

with solution

$$\begin{aligned} \phi(z) &= -\frac{s+z}{2(b_0-s)} \pm \frac{\sqrt{(s+z)^2 + 4z(b_0-s)}}{2(b_0-s)} \\ &\approx -\frac{s+z}{2b_0} \pm \frac{\sqrt{(s+z)^2+4zb_0}}{2b_0} \\ &\approx \begin{cases} \frac{z}{s+z} & (s+z)^2 \gg 4zb_0 \\ \sqrt{\frac{z}{b_0}}\left(1 + \frac{(s+z)^2}{8zb_0}\right) - \frac{s+z}{2b_0} & (s+z)^2 \ll 4zb_0 \end{cases} \end{aligned} \tag{7}$$

Since $\phi(z) = 1 - \int e^{-wz}p(w)\, dw$, $\phi(\epsilon)$ is exactly the probability that a lineage is sampled when the entire population is sampled at rate ϵ. We thus expect two regimes: if the square of the fitness cost exceeds the sampling intensity (typically at 1% or less), the probability of sampling a lineage is essentially inversely proportional to the fitness cost. The sampling probability of lineages with smaller costs effects depends less strongly on s. Their sampling mostly comes down to stochasticity independent of the fitness cost.

bioRxiv preprint doi: https://doi.org/10.1101/2023.01.30.526314; this version posted June 6, 2023. The copyright holder for this preprint (which was not certified by peer review) is the author/funder, who has granted bioRxiv a license to display the preprint in perpetuity. It is made available under aCC-BY 4.0 International license.

Growing populations

In many scenarios relevant for lineages that arise during a viral outbreak, the background population isn't constant but is undergoing a rapid exponential expansion. The background birth rate b_0 is bigger than d_0 in this case. Since the population is growing, deleterious mutations can increase in frequency deterministically and we can not send the t to infinity as before. Instead, we need to integrate

$$\partial_t \phi(z, T|t) = -z - (b - d - z)\phi(z, T|t) + b\phi(z, T|t)^2 \tag{8}$$

backwards in time starting from $\phi(z, T|T) = 0$ at $t = T$. While $\phi(z, T|t)$ is small and the quadratic term can be neglected, this is approximately solved by

$$\begin{aligned} \phi(z, T|t) &= ze^{\int_t^T (b-d-z)dt'} \int_t^T e^{-\int_\tau^T (b-d-z)dt'} d\tau \\ &= ze^{(b-d-z)(T-t)} \int_t^T e^{-(b-d-z)(T-\tau)} d\tau \\ &= ze^{(b-d-z)(T-t)} \left[1 - e^{-(b-d-z)(T-t)}\right] / (b-d-z) \\ &= \frac{z}{b-d-z} \left[e^{(b-d-z)(T-t)} - 1\right] = \frac{z}{\gamma_0 - s - z} \left[e^{\gamma_0(T-t)-(s+z)(T-t)} - 1\right] \end{aligned} \tag{9}$$

where γ_0 is the growth rate of the background population.

At longer times when $ze^{\gamma_0(T-t)} \sim 1$ and ϕ is no longer small, ϕ tends towards a constant value determined by the same quadratic equation as above. This limit is neither interesting or relevant for the present purpose, since there are very few lineages that emerged early enough to have saturated ϕ. Instead, we need to average ϕ (the linear approximation) over all the time points when the lineage could have arisen.

$$\begin{aligned} \langle \phi \rangle &\sim \int_t^T dt'\, e^{-\gamma_0(T-t')} \frac{z(e^{\gamma_0(T-t')-(s+z)(T-t')} - 1)}{(\gamma_0 - s - z)} \\ &= \int_t^T dt'\, \frac{z(e^{-(s+z)(T-t')} - e^{-\gamma_0(T-t')})}{(\gamma_0 - s - z)} \\ &\approx \begin{cases} \frac{z}{\gamma_0-s-z}\left[\frac{1}{z+s} - \frac{1}{\gamma_0}\right] & s(T-t) \gg 1 \\ \frac{z}{\gamma_0-s-z}\left[(T-t) - \frac{(s+z)(T-t)^2}{2} - \frac{1}{\gamma_0}\right] & s(T-t) < 1 \end{cases} \end{aligned} \tag{10}$$

This derivation assumed that $\gamma_0(T-t) \gg 1$, i.e. that the overall population size has expanded substantially. The most relevant fitness effects will be those with $s(T-t) > 1$, that is the fitness effect has strong effect on variant frequency, but $s < \gamma_0$ such that the variant is still spreading and can give rise to large lineages in an expanding variant. In this case, the above simplifies to

$$\langle \phi \rangle \approx \frac{z}{\gamma_0(z+s)} \tag{11}$$

In a variant that has been growing with rate γ_0 for a time $\tau = T - t$ and sampled with $z = \epsilon$, we thus expect that the number of times we observe separate mutant lineages depends on s as

$$\langle \phi \rangle \approx \frac{\epsilon}{\gamma_0(\epsilon+s)} \tag{12}$$

This has a very similar behavior as the solution for constant population size, which suggests that the overall dependence on s is robust and we can assume that the number of times a mutation is observed is inversely proportional to its effect on fitness. The same basic dependency is observed at steady state in a quasi-species [65]. In a constant population, this relationship breaks down for dense sampling $\epsilon > \sqrt{s}$. In growing population, the approximation fails if the product of fitness effect and the time over which the variant has grown, $s\tau$, is small, i.e., if the fitness cost does not affect variant frequency strongly. In these cases, there is still a dependence on s, but it is weaker.

bioRxiv preprint doi: https://doi.org/10.1101/2023.01.30.526314; this version posted June 6, 2023. The copyright holder for this preprint (which was not certified by peer review) is the author/funder, who has granted bioRxiv a license to display the preprint in perpetuity. It is made available under aCC-BY 4.0 International license.

Supplementary figures

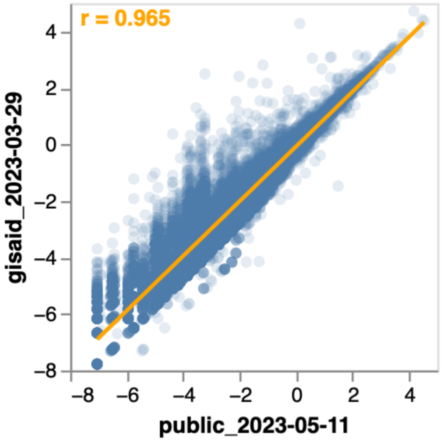

Figure S1 Correlation between amino-acid fitness estimates from the ∼7-million sequence set of all publicly available sequences as of May-11-2023, or the set of all ∼14-million sequences in GISAID as of March-29-2023. The orange text in the upper-left corner of the plot shows the Pearson correlation. The high correlation indicates that our estimates are not substantially limited by noise related to statistical sampling of mutation counts, since using another tree with twice as many sequences does not substantially alter the estimates. Note that the main text figures all use the smaller ∼7-million publicly available sequence set. See https://jbloomlab.github.io/SARS2-mut-fitness/mat_aa_fitness_correlations.html for an interactive version of this plot that also shows the correlations for other sets of publicly available sequences.

bioRxiv preprint doi: https://doi.org/10.1101/2023.01.30.526314; this version posted June 6, 2023. The copyright holder for this preprint (which was not certified by peer review) is the author/funder, who has granted bioRxiv a license to display the preprint in perpetuity. It is made available under aCC-BY 4.0 International license.

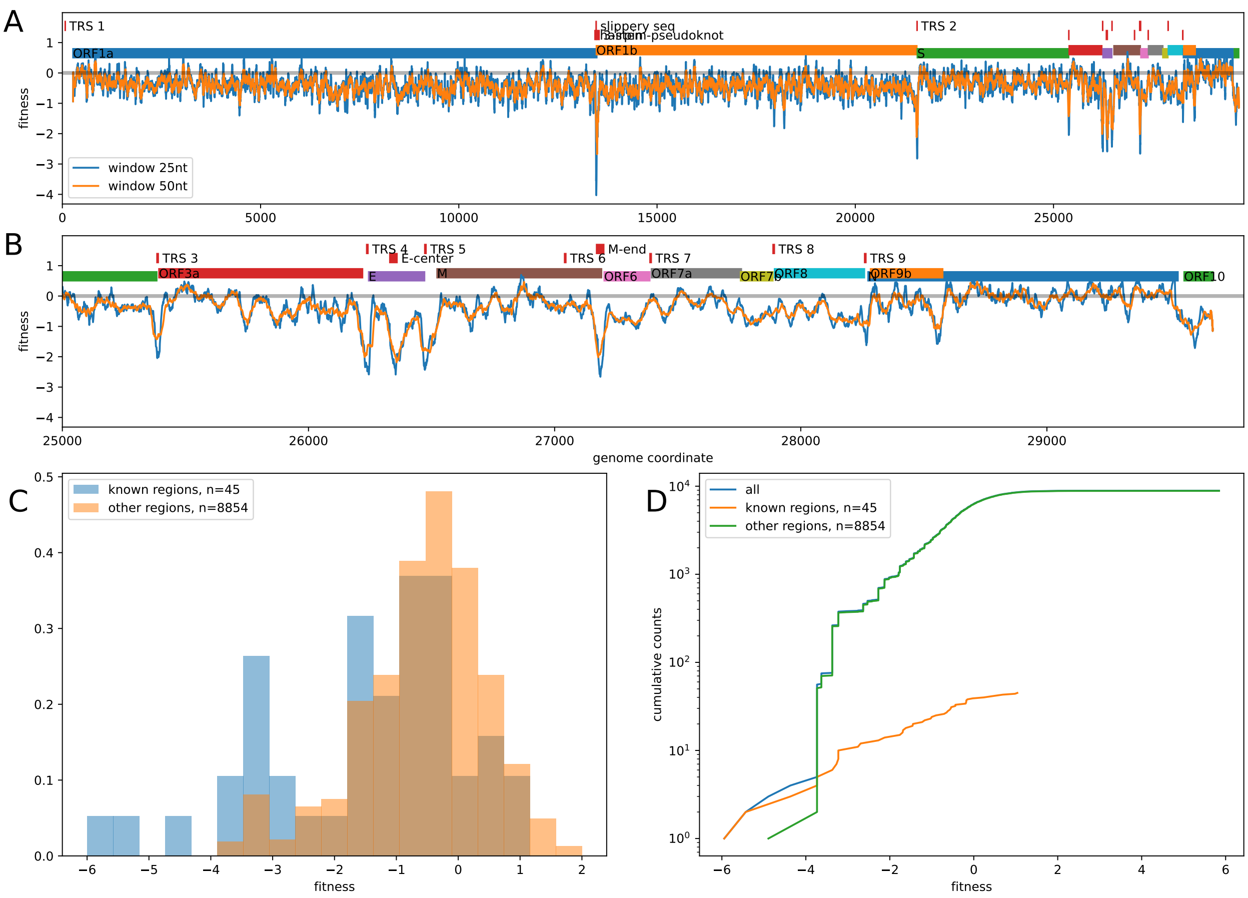

Figure S2 Non-coding constraint is concentrated in a few isolated regions in SARS-CoV-2 genome. (A), (B) The average fitness cost of synonymous mutations (do not change amino acids) in ORF1ab, S, E, M, N, and ORF3a, non-coding regions between ORFs, and all positions in ORF6, ORF7a, ORF7b, ORF8, ORF10 (as these ORFs tolerate stop codons, see Figure 3B). Panel A shows the entire genome, panel B zooms into the 3′ end of the genome. Fitness effects are averaged in sliding windows of size 25 and 50. With the exception of a small number of well defined regions, in most of the genome these averages fluctuate around zero. Regions with large fitness costs for non-coding mutations correspond to the ribosomal slippage site at the end of ORF1a, the transcription regulatory sites of S, ORF3a, E, M, and N, as well as two regions in the center of E and the end of M (see red bars in the top part of each panel) [72]. Notably, TRS sites of ORF6, ORF7a and ORF8 don't show strong signal of conservation, consistent with their tolerance of stop codons. **(C), (D)** The distribution of inferred fitness cost of four-fold degenerate sites at regions of known non-coding constraint within the protein coding genes (the ribosomal slippage and TRS sites) versus all other four-fold degenerate sites. In total, 25 four-fold degenerate sites are in these regions of known constraint, whereas 4844 four-fold degenerate sites are outside these regions. Panel C shows that a large fraction of mutations in these known regions of constraint are deleterious, while the distribution for the remaining four-fold degenerate sites is centered around 0 between -2 and +1. Furthermore, strongly deleterious mutations with fitness estimate below -3 predominantly fall in the 25 sites of known non-coding constraint. If the two additional conserved regions in E and M are included, all but one mutation outside these regions has a fitness cost estimate above -3.

bioRxiv preprint doi: https://doi.org/10.1101/2023.01.30.526314; this version posted June 6, 2023. The copyright holder for this preprint (which was not certified by peer review) is the author/funder, who has granted bioRxiv a license to display the preprint in perpetuity. It is made available under aCC-BY 4.0 International license.

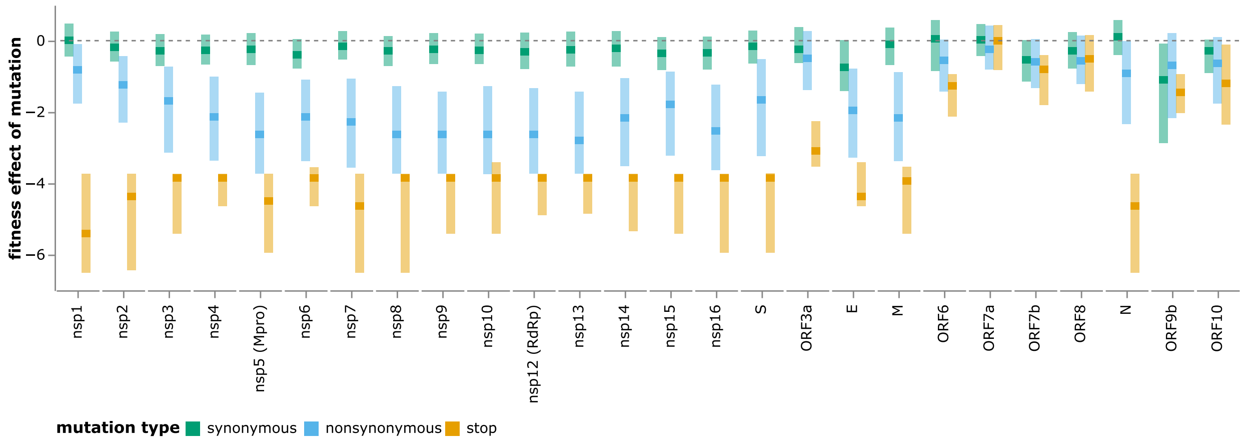

Figure S3 A version of Figure 3B but with genes ordered by position in the genome rather than extent of constraint on nonsynonymous mutations. See https://jbloomlab.github.io/SARS2-mut-fitness/effects_dist_position_order.html for an interactive version of this plot.

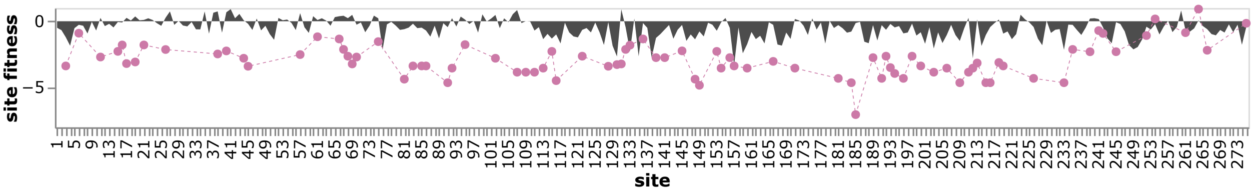

Figure S4 Effects of stop-codon and amino-acid mutations across ORF3a. The black area plot shows the mean effect of all amino-acid mutations at each site, and the purple points show the effects of stop codon mutations. There is strong selection against stop codons (negative effects) for all but the C-terminus of ORF3a, but only a few positions show strong selection against amino-acid substitutions. See https://jbloomlab.github.io/SARS2-mut-fitness/ORF3a.html for an interactive version of this plot along with zoomable heatmap of the effects of specific amino-acid substitutions.

bioRxiv preprint doi: https://doi.org/10.1101/2023.01.30.526314; this version posted June 6, 2023. The copyright holder for this preprint (which was not certified by peer review) is the author/funder, who has granted bioRxiv a license to display the preprint in perpetuity. It is made available under aCC-BY 4.0 International license.

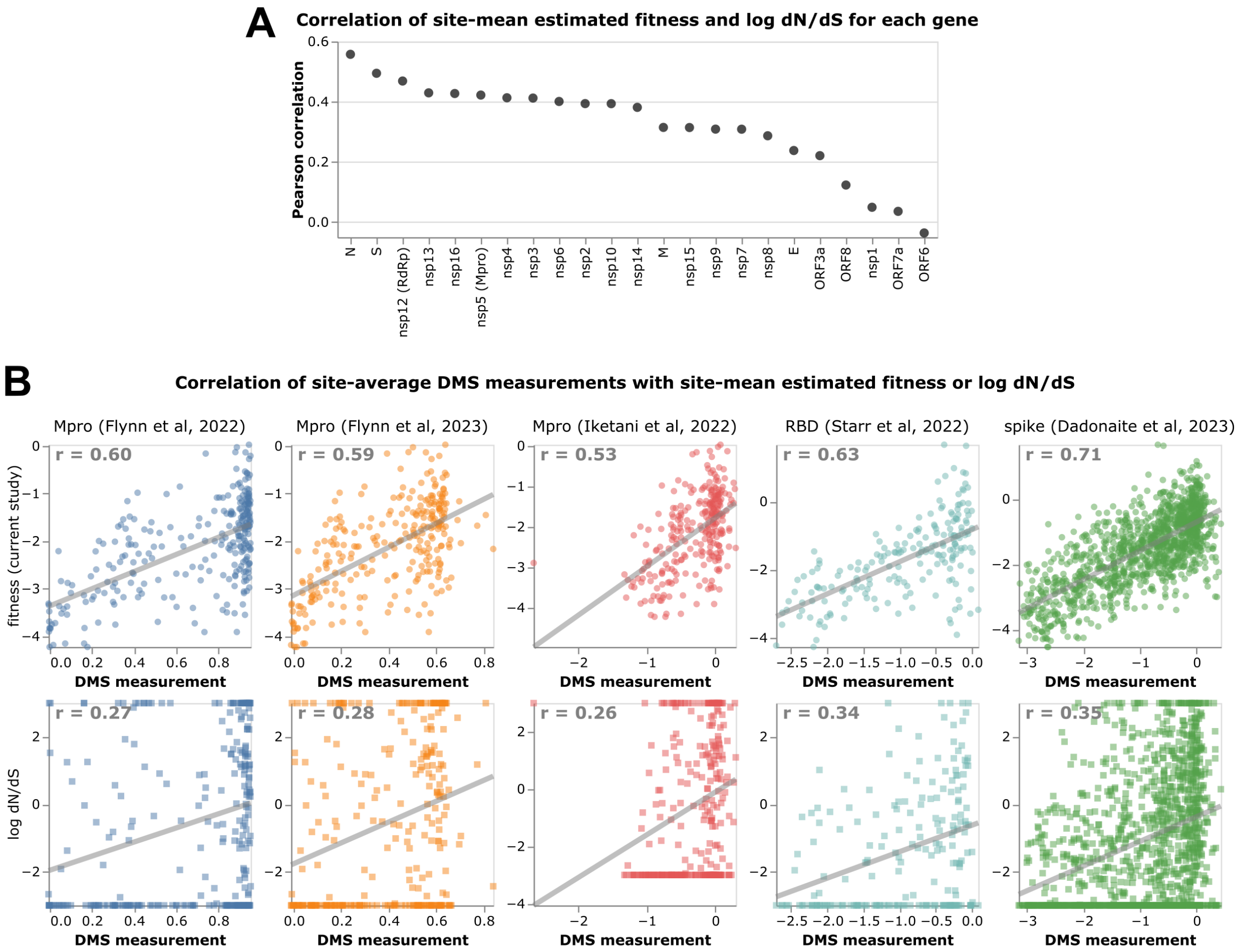

Figure S5 The site-mean of the estimated fitness effects outperforms dN/dS ratios as an indicator of functional constraint as assessed by correlation with deep mutational scanning measurements. **(A)** Pearson correlation of log dN/dS ratio at each site with the mean estimated fitness of all amino acid mutations at each site from the current study. For most genes, the log dN/dS ratio is correlated with the mean fitness effect, but the correlations are only modest (always substantially less than one). The exception is the accessory proteins which are mostly under minimal amino-acid level selection (Figure 3B) and only show a weak correlation. **(B)** Correlation of the log dN/dS ratio or the mean estimated fitness effect at each site with the mean impact of amino-acid mutations at that site as experimentally measured by deep mutational scanning. The numbers in the upper right of each panel give the Pearson correlation. In every case, the mean estimated fitness effect (top row) is much better correlated with the experimental measurements than the log dN/dS (bottom row). The dN/dS ratios are taken from https://github.com/spond/SARS-CoV-2-variation. See https://jbloomlab.github.io/SARS2-mut-fitness/dnds_corr.html for an interactive version of this plot.

bioRxiv preprint doi: https://doi.org/10.1101/2023.01.30.526314; this version posted June 6, 2023. The copyright holder for this preprint (which was not certified by peer review) is the author/funder, who has granted bioRxiv a license to display the preprint in perpetuity. It is made available under aCC-BY 4.0 International license.

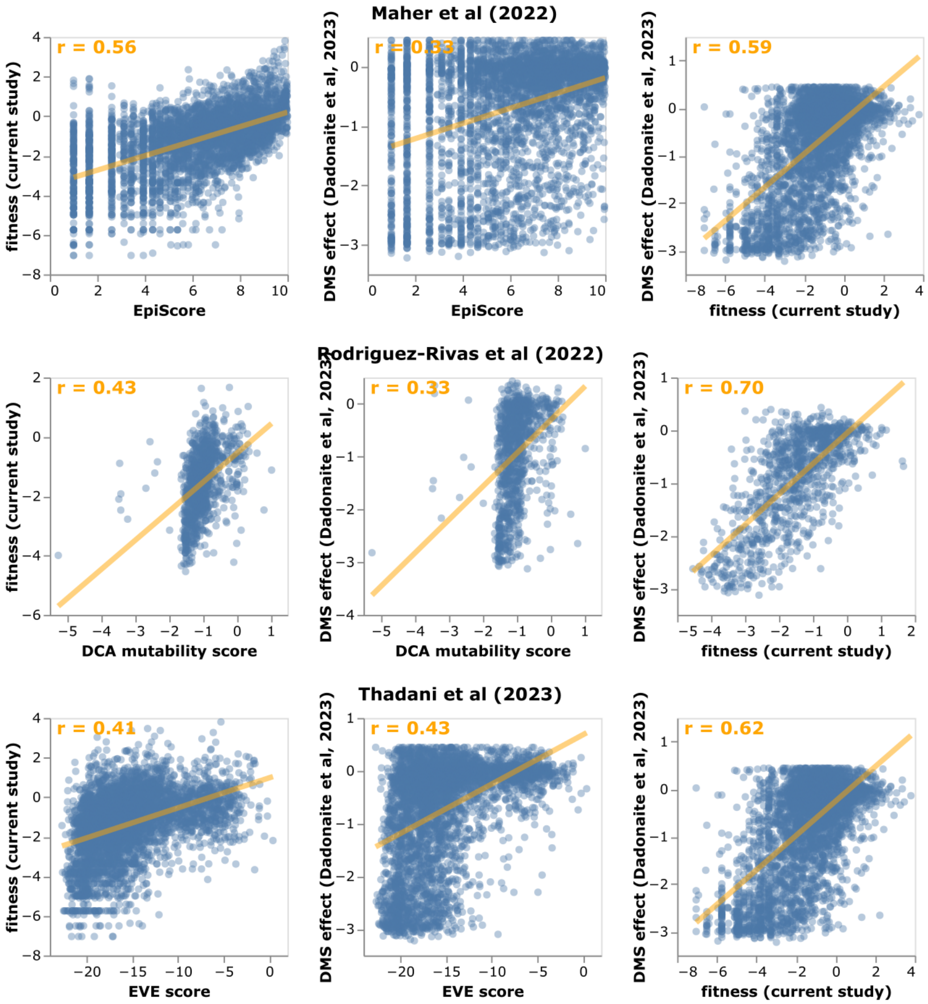

Figure S6 The mutation effect estimates from the current study are substantially better correlated with deep mutational scanning measurements for spike than predicted mutation effects made using three other approaches. Each row shows the correlation of the fitness estimates from the current study and full-spike deep mutational scanning measurements versus values from another study. The Maher et al (2022) and Thadani et (2023) studies make predictions for individual amino-acids, so those correlations are with the mutation-level fitness estimates and deep mutational scanning measurements. The Rodriguez-Rivas et al (2022) study only reports site-level predictions, so those correlations are with the site mean fitness estimates and deep mutational scanning measurements. Each row only shows mutations or sites with a reported value from all three approaches. The numbers in the upper right give the Pearson correlation. See https://jbloomlab.github.io/SARS2-mut-fitness/comparator_corr.html for an interactive version of this plot.

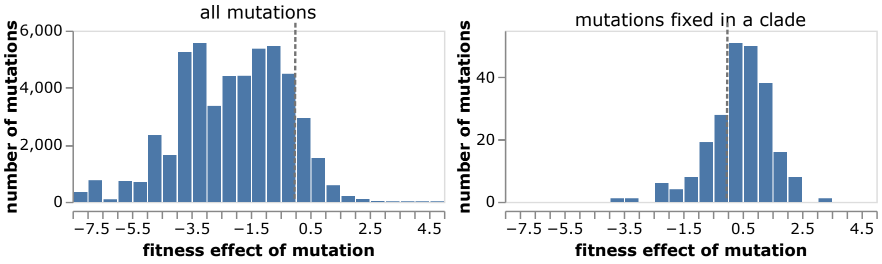

Figure S7 Distribution of fitness effects of all amino-acid mutations relative to Wuhan-Hu-1, and just those mutations that fixed in at least one clade of SARS-CoV-2 (using the Nextstrain clade definitions). The vertical dashed line at zero indicates the effect of a neutral mutation. See https://jbloomlab.github.io/SARS2-mut-fitness/clade_fixed_muts_hist.html for an interactive version of this plot that allows adjustment of the minimum expected count threshold.

bioRxiv preprint doi: https://doi.org/10.1101/2023.01.30.526314; this version posted June 6, 2023. The copyright holder for this preprint (which was not certified by peer review) is the author/funder, who has granted bioRxiv a license to display the preprint in perpetuity. It is made available under aCC-BY 4.0 International license.

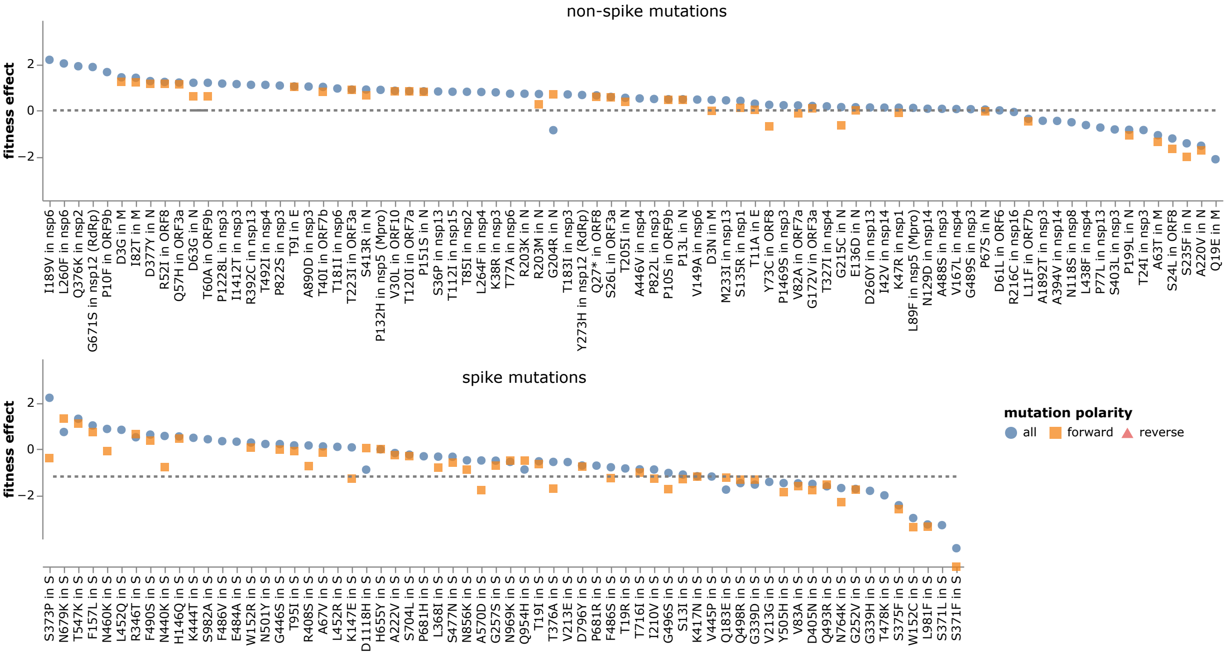

Figure S8 Effects of individual mutations that fixed in at least one clade of SARS-CoV-2, faceted by whether they are in spike or another protein. "Mutation polarity" indicates if the point shows the effect of the mutation estimated using all viral clades (including those that have fixed the mutation), or just from direct forward occurrences of the mutation in clades in which it has not yet fixed. Some mutations are estimated to be more favorable when including clades in which they have fixed (blue circles) in addition to just clades in which it has not yet fixed (orange squares)—when this occurs, it suggests epistatic entrenchment of the mutations [38, 60]. Note that clades in which a mutation has already fixed contribute to estimates of its fitness via estimates of the effect of its reversion and via estimates of the effects of mutations to other amino acids at the same site. See https://jbloomlab.github.io/SARS2-mut-fitness/clade_fixed_muts.html for an interactive version of this plot.

bioRxiv preprint doi: https://doi.org/10.1101/2023.01.30.526314; this version posted June 6, 2023. The copyright holder for this preprint (which was not certified by peer review) is the author/funder, who has granted bioRxiv a license to display the preprint in perpetuity. It is made available under aCC-BY 4.0 International license.

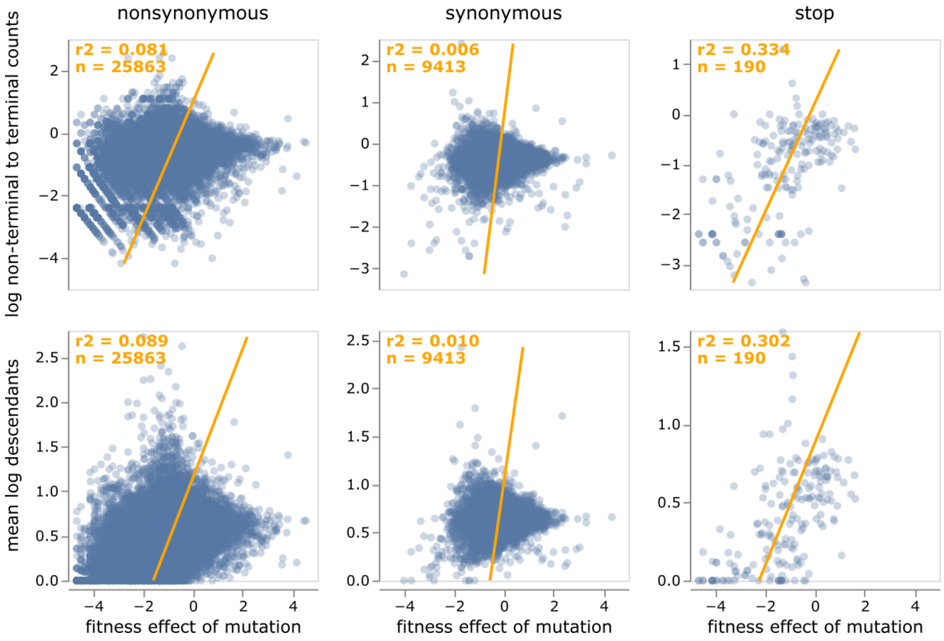

Figure S9 Relationship between fitness effects of mutations and two measures of the number of descendants. At top is shown the log ratio of counts of the mutation on non-terminal (internal) to terminal (tip) branches; larger values indicate mutations more likely to be found in viruses that leave descendants. At bottom is shown the mean log number of tip descendants that share all the mutations on each branch containing the mutation of interest; larger values again indicate mutations more likely to be found in viruses that leave more descendants. Each point is an amino-acid mutation, the orange line is a least-squares regression, and the orange text in the upper left give the number of mutations and the Pearson correlation coefficient. This plot shows only mutations with at least 10 expected counts and 5 actual counts. See https://jbloomlab.github.io/SARS2-mut-fitness/fitness_vs_terminal.html for an interactive version of this plot that allows filtering by the number of actual or expected counts, or by gene. The number of descendants is calculated using the “leaves_sharing_mutations” variable of the UShER mutation-annotated tree.

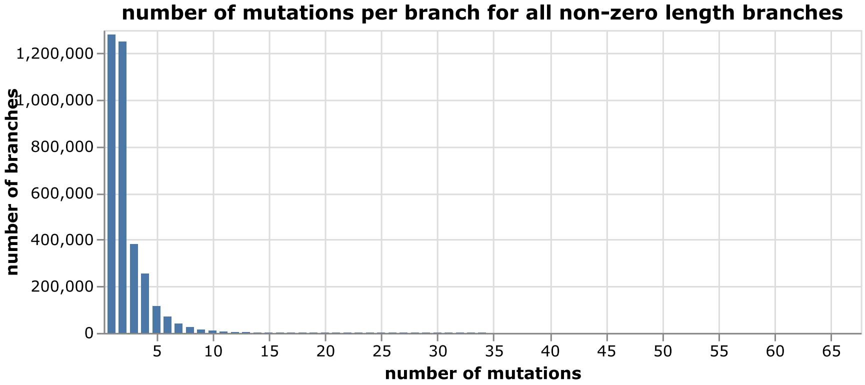

Figure S10 Number of mutations per branch on all branches of the SARS-CoV-2 tree with at least one mutation. The analysis used here only considers mutations on branches with four or fewer mutations and so excludes the small fraction of highly mutated branches.
