# Supplementary material for: Fitness effects of mutations to SARS-CoV-2 proteins
Source: bioRxiv. 2023 Jan 31:2023.01.30.526314. Preprint. [Version 1] doi: 10.1101/2023.01.30.526314 (PMC9915511; doi:10.1101/2023.01.30.526314)
Supplement: Supplementary file 1 [file NIHPP2023.01.30.526314v1-supplement-1.pdf]

bioRxiv preprint doi: https://doi.org/10.1101/2023.01.30.526314; this version posted January 31, 2023. The copyright holder for this preprint (which was not certified by peer review) is the author/funder, who has granted bioRxiv a license to display the preprint in perpetuity. It is made available under aCC-BY 4.0 International license.

Supplementary appendix deriving relationship between fitness cost and ratio of expected to actual counts

With millions of SARS-CoV-2 sequences shared publicly, almost all mutations that are tolerated by the virus are observed dozens to hundreds of times. Where on the tree and how often on the tree we observe specific mutations has information about the effects of these mutations on viral spread. The mutation rate depends on the nucleotides involved and possibly on the sequence context and other viral determinants, but for the purpose of this derivation, we will assume the neutral rate μ is known. If the mutation is neutral, the total number of times the mutation is observed on the tree is μT, where T is the total length of the tree (assuming that the mutation never reached high frequency which is true for almost all mutations, particularly when mutations are counted on a per-clade basis relative too the clade founder as done above).

If a mutation reduces fitness, the lineages descending from branches on which this mutation happened will spread more slowly than those without this mutation. As a result, the down-stream subclades are smaller and more short lived, which in turn means that they will be less likely to be sampled and represented in the tree. To infer a mutation's effect on fitness, we need to calculate how the probability of observation depends on this fitness effect.

For a mutation to be represented in the tree, one of its descendants has to be sampled and sequenced. If the total number of descendants is w and the sampling fraction is ϵ, the probability that the mutation is present in the tree is

$$P = 1 - e^{-w\epsilon} \tag{1}$$

W is a random number that depends on the realization of the transmission process, which is commonly modeled by a branching process with birth rate b and death rate d. The death rate here corresponds to clearing an infection, the birth rate to onward transmission. The latter is affected by the fitness cost of the mutation.

To obtain insight how the probability of observing a lineage depends on parameters, we calculate the probability $p(w, T|t)$ that a lineage had an integrated size $w = \int_t^T k(t')\, dt'$, where t is the birth time of the lineage, T is the current time, and $k(t')$ is the size of the lineage at time t'. To calculate $p(w, T|k)$, we generalize it slightly to $p(W, T|k, t)$, where k is the number of individuals at the start time t. This quantity obeys the following "first-step" equation:

$$-(\partial_t - k\partial_w)p(w, T|k, t) = -k(b+d)p(w, T|k, t) + kbp(w, T|k+1, t) + kdp(w, T|k-1, t) \tag{2}$$

We will solve for the Laplace transform $\hat{p}(z, T|k, t) = \int_0^\infty dw\, e^{-wz} p(w, T|k, t) = \hat{p}^k(z, T|1, t)$. Using the following identity for the derivative of the Laplace transform

$$\int_0^\infty e^{-wz}\partial_w p\, dw = [e^{-wz}p]_0^\infty - \int_0^\infty p\partial_w e^{-wz}\, dw = 0 + z\int_0^\infty pe^{-wz} = z\hat{p} \tag{3}$$

and setting $k = 1$, we have

$$-\partial_t \hat{p}(z, T|t) = -(b+d+z)\hat{p}(z, T|t) + b\hat{p}^2(z, T|t) + d \tag{4}$$

This simplifies further to if we substitute $\phi(z, T|t) = 1 - \hat{p}(z, T|t)$.

$$\begin{aligned}\partial_t\phi(z, T|t) &= -(b+d+z)(1-\phi(z, T|t)) + b(1-\phi(z, T|t))^2 + d \\ &= -z - (b-d-z)\phi(z, T|t) + b\phi(z, T|t)^2\end{aligned} \tag{5}$$

where it is important to note that the derivative is with respect to the first time point and the interval $T - t$ is shrinking with increasing t.

Constant birth and death rate

If the fitness effect of the mutation in question is detrimental and the overall population is constant (background $b_0 = d_0$), all mutant lineages will eventually die out and we can consider large $T - t$ and the long time asymptotic $\partial_t\phi(z, T|t) = 0$. Further define $b = b_0 - s$ and $d = d_0$ where s is the fitness cost of the mutation (so larger values indicate a greater fitness cost). The steady state generating function is then

$$0 = -z - (b-d-z)\phi(z) + b\phi(z)^2 \tag{6}$$

with solution

$$\begin{aligned}\phi(z) &= -\frac{s+z}{2(b_0-s)} \pm \frac{\sqrt{(s+z)^2 + 4z(b_0-s)}}{2(b_0-s)} \\ &\approx -\frac{s+z}{2b_0} \pm \frac{\sqrt{(s+z)^2+4zb_0}}{2b_0} \\ &\approx \begin{cases} \frac{z}{s+z} & (s+z)^2 \gg 4zb_0 \\ \sqrt{\frac{z}{b_0}}\left(1 + \frac{(s+z)^2}{8zb_0}\right) - \frac{s+z}{2b_0} & (s+z)^2 \ll 4zb_0 \end{cases}\end{aligned} \tag{7}$$

Since $\phi(z) = 1 - \int e^{-wz}p(w)\, dw$, $\phi(\epsilon)$ is exactly the probability that a lineage is sampled when the entire population is sampled at rate ϵ. We thus expect two regimes: if the square of the fitness cost exceeds the sampling intensity (typically at 1% or less), the probability of sampling a lineage is essentially inversely proportional to the fitness cost. The sampling probability of lineages with smaller costs effects depends less strongly on s. Their sampling mostly comes down to stochasticity independent of the fitness cost.

bioRxiv preprint doi: https://doi.org/10.1101/2023.01.30.526314; this version posted January 31, 2023. The copyright holder for this preprint (which was not certified by peer review) is the author/funder, who has granted bioRxiv a license to display the preprint in perpetuity. It is made available under aCC-BY 4.0 International license.

Growing populations

In many scenarios relevant for lineages that arise during a viral outbreak, the background population isn't constant but is undergoing a rapid exponential expansion. The background birth rate b_0 is bigger than d_0 in this case. Since the population is growing, deleterious mutations can increase in frequency deterministically and we can not send the t to infinity as before. Instead, we need to integrate

$$\partial_t \phi(z, T|t) = -z - (b - d - z)\phi(z, T|t) + b\phi(z, T|t)^2 \tag{8}$$

backwards in time starting from $\phi(z, T|T) = 0$ at $t = T$. While $\phi(z, T|t)$ is small and the quadratic term can be neglected, this is approximately solved by

$$\begin{aligned}
\phi(z, T|t) &= z e^{\int_t^T (b-d-z)dt'} \int_t^T e^{-\int_\tau^T (b-d-z)dt'} d\tau \\
&= z e^{(b-d-z)(T-t)} \int_t^T e^{-(b-d-z)(T-\tau)} d\tau \\
&= z e^{(b-d-z)(T-t)} \left[1 - e^{-(b-d-z)(T-t)}\right] / (b - d - z) \\
&= \frac{z}{b-d-z}\left[e^{(b-d-z)(T-t)} - 1\right] = \frac{z}{\gamma_0 - s - z}\left[e^{\gamma_0(T-t)-(s+z)(T-t)} - 1\right]
\end{aligned} \tag{9}$$

where γ_0 is the growth rate of the background population.

At longer times when $ze^{\gamma_0(T-t)} \sim 1$ and ϕ is no longer small, ϕ tends towards a constant value determined by the same quadratic equation as above. This limit is neither interesting or relevant for the present purpose, since there are very few lineages that emerged early enough to have saturated ϕ. Instead, we need to average ϕ (the linear approximation) over all the time points when the lineage could have arisen.

$$\begin{aligned}
\langle \phi \rangle &\sim \int_t^T dt' \, e^{-\gamma_0(T-t')} \frac{z(e^{\gamma_0(T-t')-(s+z)(T-t')} - 1)}{(\gamma_0 - s - z)} \\
&= \int_t^T dt' \, \frac{z(e^{-(s+z)(T-t')} - e^{-\gamma_0(T-t')})}{(\gamma_0 - s - z)} \\
&\approx \begin{cases} \frac{z}{\gamma_0 - s - z}\left[\frac{1}{z+s} - \frac{1}{\gamma_0}\right] & s(T-t) \gg 1 \\ \frac{z}{\gamma_0 - s - z}\left[(T-t) - \frac{(s+z)(T-t)^2}{2} - \frac{1}{\gamma_0}\right] & s(T-t) < 1 \end{cases}
\end{aligned} \tag{10}$$

This derivation assumed that $\gamma_0(T-t) \gg 1$, i.e. that the overall population size has expanded substantially. The most relevant fitness effects will be those with $s(T-t) > 1$, that is the fitness effect has strong effect on variant frequency, but $s < \gamma_0$ such that the variant is still spreading and can give rise to large lineages in an expanding variant. In this case, the above simplifies to

$$\langle \phi \rangle \approx \frac{z}{\gamma_0(z+s)} \tag{11}$$

In a variant that has been growing with rate γ_0 for a time $\tau = T - t$ and sampled with $z = \epsilon$, we thus expect that the number of times we observe separate mutant lineages depends on s as

$$\langle \phi \rangle \approx \frac{\epsilon}{\gamma_0(\epsilon + s)} \tag{12}$$

This has a very similar behavior as the solution for constant population size, which suggests that the overall dependence on s is robust and we can assume that the number of times a mutation is observed is inversely proportional to its effect on fitness. The same basic dependency is observed at steady state in a quasi-species [55]. In a constant population, this relationship breaks down for dense sampling $\epsilon > \sqrt{s}$. In growing population, the approximation fails if the product of fitness effect and the time over which the variant has grown, $s\tau$, is small, i.e., if the fitness cost does not affect variant frequency strongly. In these cases, there is still a dependence on s, but it is weaker.

bioRxiv preprint doi: https://doi.org/10.1101/2023.01.30.526314; this version posted January 31, 2023. The copyright holder for this preprint (which was not certified by peer review) is the author/funder, who has granted bioRxiv a license to display the preprint in perpetuity. It is made available under aCC-BY 4.0 International license.

Supplementary figures

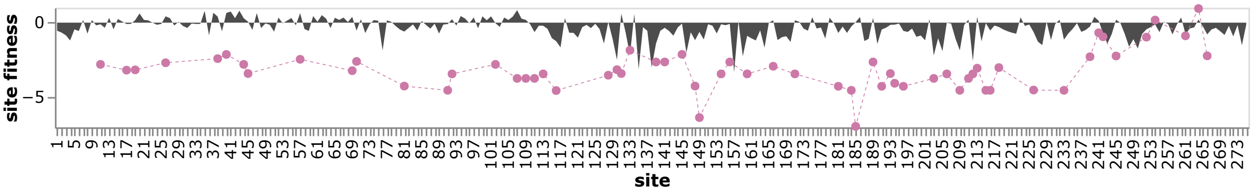

Figure S1 Effects of stop-codon and amino-acid mutations across ORF3a. The black area plot shows the mean effect of all amino-acid mutations at each site, and the purple points show the effects of stop codon mutations. There is strong selection against stop codons (negative effects) for all but the C-terminus of ORF3a, but only a few positions show strong selection against amino-acid substitutions. This plot shows only mutations with 20 expected counts. See https://jbloomlab.github.io/SARS2-mut-fitness/ORF3a.html for an interactive version of this plot along with zoomable heatmap of the effects of specific amino-acid substitutions.

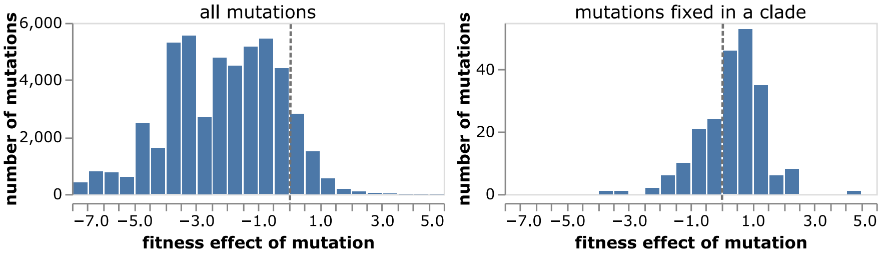

Figure S2 Distribution of fitness effects of all amino-acid mutations relative to Wuhan-Hu-1, and just those mutations that fixed in at least one clade of SARS-CoV-2 (using the Nextstrain clade definitions). The vertical dashed line at zero indicates the effect of a neutral mutation. See https://jbloomlab.github.io/SARS2-mut-fitness/clade_fixed_muts_hist.html for an interactive version of this plot that allows adjustment of the minimum expected count threshold.

bioRxiv preprint doi: https://doi.org/10.1101/2023.01.30.526314; this version posted January 31, 2023. The copyright holder for this preprint (which was not certified by peer review) is the author/funder, who has granted bioRxiv a license to display the preprint in perpetuity. It is made available under aCC-BY 4.0 International license.

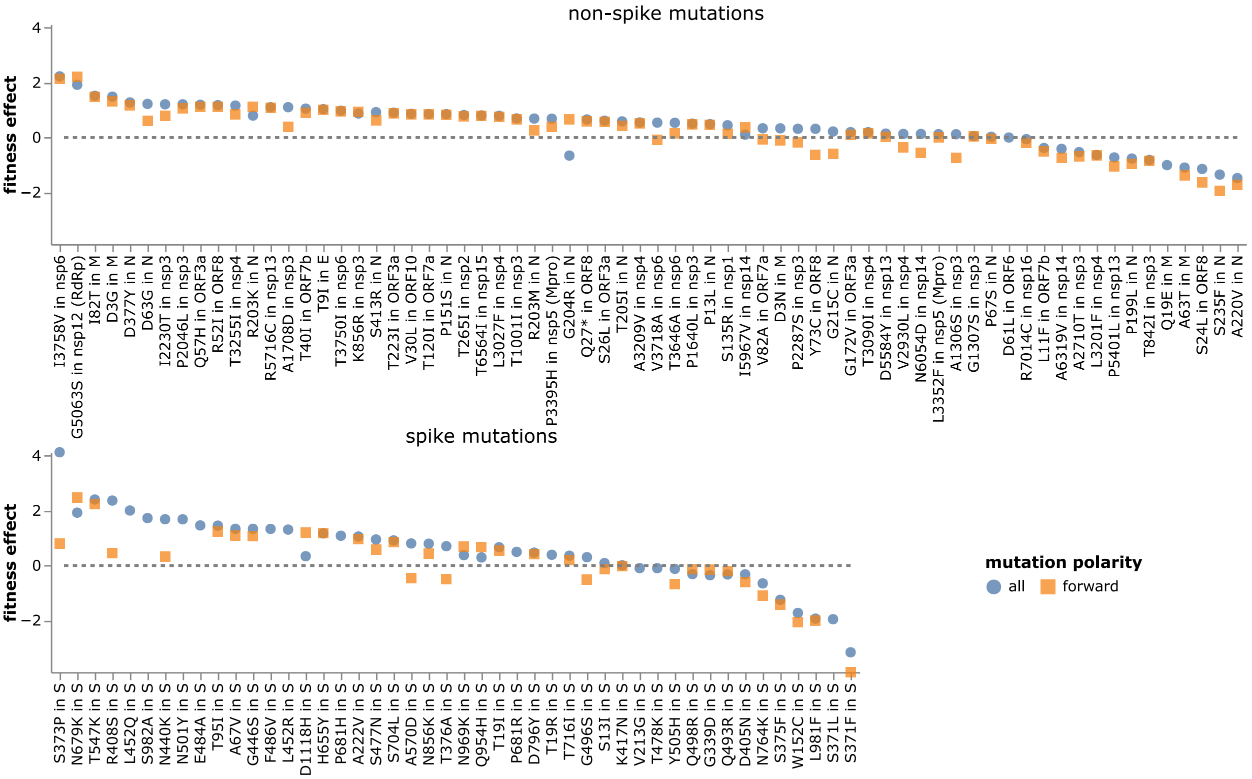

Figure S3 Effects of individual mutations that fixed in at least one clade of SARS-CoV-2, faceted by whether they are in spike or another protein. "Mutation polarity" indicates if the point shows the effect of the mutation estimated using all viral clades (including those that have fixed the mutation), or just from direct forward occurrences of the mutation in clades in which it has not yet fixed. Some mutations are estimated to be more favorable when including clades in which they have fixed (blue circles) in addition to just clades in which it has not yet fixed (orange squares)—when this occurs, it suggests epistatic entrenchment of the mutations [38, 50]. Note that clades in which a mutation has already fixed contribute to estimates of its fitness via estimates of the effect of its reversion and via estimates of the effects of mutations to other amino acids at the same site. See https://jbloomlab.github.io/SARS2-mut-fitness/clade_fixed_muts.html for an interactive version of this plot.

bioRxiv preprint doi: https://doi.org/10.1101/2023.01.30.526314; this version posted January 31, 2023. The copyright holder for this preprint (which was not certified by peer review) is the author/funder, who has granted bioRxiv a license to display the preprint in perpetuity. It is made available under aCC-BY 4.0 International license.

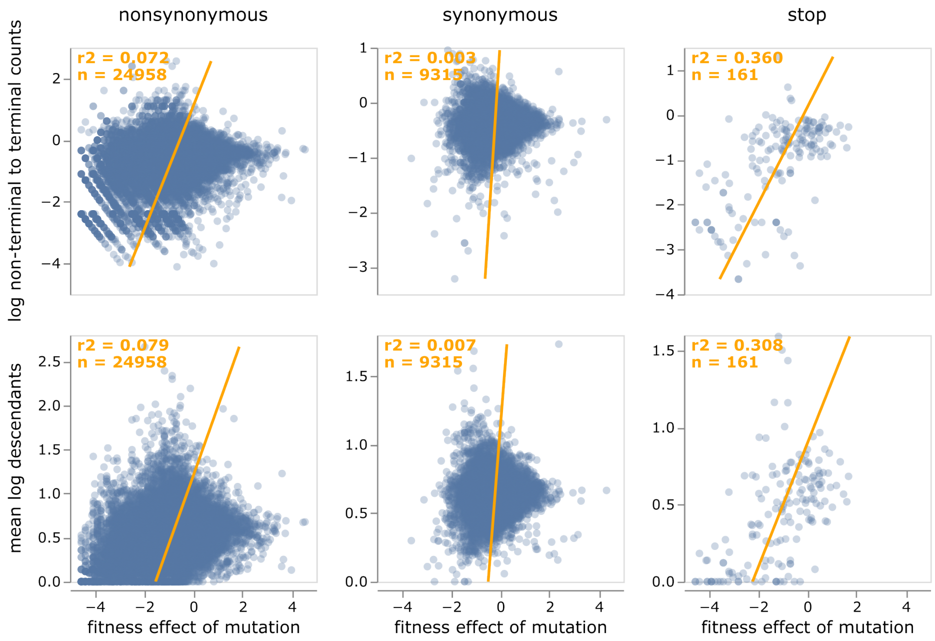

Figure S4 Relationship between fitness effects of mutations and two measures of the number of descendants. At top is shown the log ratio of counts of the mutation on non-terminal (internal) to terminal (tip) branches; larger values indicate mutations more likely to be found in viruses that leave descendants. At bottom is shown the mean log number of tip descendants that share all the mutations on each branch containing the mutation of interest; larger values again indicate mutations more likely to be found in viruses that leave more descendants. Each point is an amino-acid mutation, the orange line is a least-squares regression, and the orange text in the upper left give the number of mutations and the Pearson correlation coefficient. This plot shows only mutations with at least 10 expected counts and 5 actual counts. See https://jbloomlab.github.io/SARS2-mut-fitness/fitness_vs_terminal.html for an interactive version of this plot that allows filtering by the number of actual or expected counts, or by gene. The number of descendants is calculated using the "leaves_sharing_mutations" variable of the UShER mutation-annotated tree.
